# Geraniol Ameliorates Doxorubicin-Mediated Kidney Injury through Alteration of Antioxidant Status, Inflammation, and Apoptosis: Potential Roles of NF-κB and Nrf2/Ho-1

**DOI:** 10.3390/nu14081620

**Published:** 2022-04-13

**Authors:** Abdullah F. AlAsmari, Nemat Ali, Metab Alharbi, Faleh Alqahtani, Fawaz Alasmari, Daad Almoqbel, Mohammed AlSwayyed, Abdulrahman Alshammari, Mohammed M. Alanazi, Ali Alhoshani, Naif O. Al-Harbi

**Affiliations:** 1 Department of Pharmacology and Toxicology, College of Pharmacy, King Saud University, Riyadh 11451, Saudi Arabia; nali1@ksu.edu.sa (N.A.); mesalharbi@ksu.edu.sa (M.A.); afaleh@ksu.edu.sa (F.A.); ffalasmari@ksu.edu.sa (F.A.); daadalmoqbel@gmail.com (D.A.); abdalshammari@ksu.edu.sa (A.A.); momalanazi@ksu.edu.sa (M.M.A.); ahoshani@ksu.edu.sa (A.A.); nharbi1@ksu.edu.sa (N.O.A.-H.); 2 Department of Pathology, College of Medicine, King Saud University, Riyadh 11451, Saudi Arabia; malswayyed@ksu.edu.sa

**Keywords:** doxorubicin, inflammation, oxidative stress, apoptosis, kidney injury, geraniol

## Abstract

Doxorubicin-mediated kidney impairment is a serious problem in cancer treatment. Accordingly, this work investigated the ability of geraniol to modulate doxorubicin-induced kidney damage using a rat model. Rats were randomly assigned to four groups: control, doxorubicin (20 mg/kg, intraperitoneal, i.p.), doxorubicin plus 100 mg/kg of geraniol, and doxorubicin plus 200 mg/kg of geraniol. A single doxorubicin injection triggered kidney impairment, as evidenced by the altered serum creatinine, blood urea nitrogen, and albumin values; it also caused histological changes in the kidney architecture. Additionally, doxorubicin enhanced lipid peroxidation while lowering reduced glutathione, catalase activity, and the expression of glutathione peroxidase and superoxide dismutase. Interestingly, pre-treatment with geraniol rescued doxorubicin-induced alterations in kidney antioxidant parameters, enzymatic activity, and the expression of inflammatory and apoptosis-mediating gene and proteins. Moreover, prophylactic treatment with geraniol preserved most kidney histological characteristics in a dose-dependent manner. These findings support that geraniol could protect against doxorubicin-mediated kidney dysfunction. However, further research is needed to clarify the mechanisms of geraniol’s protective effects against doxorubicin-mediated kidney dysfunction.

## 1. Introduction

Since its discovery, the anthracycline anti-cancer medication doxorubicin (Dox) has been extensively utilized against solid tumors and hematological malignancies [1,2,3,4]. Among its most common side effects is nephrotoxicity, described as renal dysfunction with reduced filtration, reabsorption, and excretion, which is associated with significant risk of morbidity and mortality. Around 60% of cancer patients who receive chemotherapy develop nephrotoxicity, restricting the therapeutic effectiveness of Dox [5,6]. While the fundamental processes that cause Dox-mediated nephrotoxicity remain unknown, the literature suggests that probable contributors include oxidative stress, inflammation, and apoptosis. In particular, evidence indicates that reducing Dox-associated oxidative stress, inflammation, and apoptotic events may help to reduce the drug’s renal toxicity [7,8,9,10,11,12,13].

Nuclear factor (erythroid-derived 2)-like 2 (Nrf2) is a redox-sensitive transcriptional factor with antioxidant, anti-inflammatory, and cytoprotective abilities that sustain cellular defense systems. Upon oxidative stress, Nrf2 releases its cytoplasmic inhibitor protein, which enters the nucleus and activates several genes involved in antioxidant defense, such as heme oxygenase-1 (Ho-1), glutathione peroxidase (GPx), catalase (CAT), and superoxide dismutase (SOD) [14,15,16,17,18,19]. Treatment with Dox has been shown to reduce both mRNA and protein expressions of Nrf2 and, hence, that of downstream antioxidant genes and proteins, leading to kidney toxicity [20,21].

Nuclear factor-κB (NF-κB) is a transcriptional factor that regulates the expression of many inflammatory genes; as the central figure of the NF-kB pathway, which is activated by reactive oxygen species (ROS), it can trigger inflammatory response and resultant tissue injury [8,22,23]. Numerous studies have reported Dox to cause inflammation and increase production of pro-inflammatory cytokines such as interleukin-6 (IL-6), interleukin-1 beta (IL-1β), and tumor necrosis factor alpha (TNF-α) [11,13]. In addition, increased ROS production after Dox-mediated kidney damage has been shown to play a key role in activating the intrinsic apoptotic pathway through mitochondrial instability. Key determinants of this pathway are the mitochondrial-associated proteins Bax and Bcl-2; a balanced ratio of Bcl-2 to Bax prevents apoptosis, while imbalance leads to increased membrane permeability and leakage of cytochrome c into the cytosol, which activates caspase-9 (Casp-9) and caspase-3 (Casp-3). Such activation generally leads to DNA fragmentation and cell death [11,24,25,26,27,28,29].

It is of great interest to maximize the clinical use of chemotherapeutic drugs while minimizing their negative effects. Accordingly, research efforts have been undertaken to investigate the use of various agents in conjunction with Dox to ameliorate its adverse effects [13,30,31]. Herbal extracts and their bioactive ingredients have long been recognized for their ability to reduce drug-mediated toxicity. Geraniol is an acyclic monoterpene alcohol found in almost all essential oils, such as those from ginger, rose, orange, lavender, and lemon [32,33]. Numerous studies have reported geraniol to have various beneficial properties, such as anti-ulcer [34], anti-cancer [35], anti-depressant [36], anti-inflammatory [33], and, furthermore, it can ease diabetic nephropathy [37].

To the best of our knowledge, there is currently no specific information on the preventive effects of geraniol against Dox-mediated nephrotoxicity. Hence, we studied the effects and probable mechanisms of the action of geraniol on injury caused by Dox through the NF-kB and Nrf2/Ho-1 signaling pathways in the kidneys of Wistar rats.

## 2. Material and Methods

### 2.1. Animals

Male Wistar rats were procured from the King Saud University (KSU) animal care center in Riyadh, Saudi Arabia. The animals were maintained in controlled conditions, such as they were kept at room temperature (25 ± 1 °C) with a 12 h light/dark cycle and had unrestricted access to water and a standard diet as authorized by the KSU Local Institutional Study Ethics Committee (REC) under authorization number KSU-SE-19-122.

### 2.2. Experimental Design

This investigation examined a total of 32 male Wistar rats weighing 190–210 g, allocated into four groups. The rats were given a week to adapt to the environment in advance of the study. The vehicle group, also known as the control group, was comprised of rats who were given an oral formulation of normal saline. Group II consisted of rats given Dox (20 mg/kg i.p. single dosage) on day 17 [11]. Group III and IV rats were given prophylactic doses of geraniol (orally) at 100 and 200 mg/kg, respectively, for 18 days, and on day 17 were, likewise, subjected to Dox (20 mg/kg, i.p.). On day 18, the rats were euthanized using a ketamine/xylazine combination in a controlled environment. Blood was collected and serum isolated, and both kidneys were removed and immediately flash-frozen in liquid nitrogen. Biochemical, gene, and protein expression assays were carried out on the frozen tissues. For histological examination, kidney tissue samples were washed in PBS and then preserved in a 4% formaldehyde solution. Throughout the investigation, there was no indication of distress or death in any experimental animal.

### 2.3. Determination of Kidney Function Markers

To isolate the serum from the blood collected at the time of sacrifice, blood samples were centrifuged for 10 min at 2000× *g* in a pre-cooled centrifuge. The obtained serum was then analyzed to quantify albumin, blood urea nitrogen (BUN), and creatinine. Values of kidney function markers were calculated using the Siemens Autoanalyzer Dimension^®^ RXL MAXTM, Siemens, Washington, DC, USA.

### 2.4. Evaluation of Lipid Peroxidation

Lipid peroxidation was evaluated in kidney tissues as described previously by Ohkawa et al., with slight modifications [38].

### 2.5. Quantification of Reduced Glutathione

Levels of reduced glutathione (GSH) in renal post-mitochondrial supernatant (PMS) were measured according to the method described by Sedlak and Lindsay, with minor modifications [39].

### 2.6. Quantification of Activity of Catalase

The activity of CAT in renal PMS was determined according to the method described by Claiborne, with minor modification [40].

### 2.7. Gene Expression Analysis (RT-qPCR)

The TRIzol™ reagent (Thermo Scientific, Waltham, MA, USA) was utilized to extract the total RNA from the kidney tissues according to the manufacturer’s guidelines. The purity and quantity of extracted RNA specimens were determined using a NanoDrop™ 8000 Spectrophotometer (Thermo Scientific, USA). The isolated RNA was then reverse transcribed into cDNA using cDNA Synthesis SuperMix (Bimake, Houston, TX, USA), and gene expression was quantified with SYBR Green Master Mix (Bimake, Houston, TX, USA) on the Applied Biosystems 7500 Fast Real-Time PCR System. The ΔΔCt approach was then used to determine the relative expression of various genes among the groups, with *GAPDH* employed as the housekeeping gene. Table 1 lists the primer sequences utilized in this study (IDT, Leuven, Belgium).

### 2.8. Immunoblot Analysis

Western blot analysis was carried out as described by Alasmari et al. [41]. Briefly, isolated proteins (30–50 μg) were electrophoresed on SDS-PAGE gels and then transferred onto PVDF membranes. After blocking with 5% nonfat dry milk for 60 min, blots were probed overnight at 4 °C with selective primary antibodies (against Ho-1, Nrf2, TNF-α, IL6, NF-κB-p65, cleaved-caspase-3, Bcl-2, Bax, and GAPDH, dilution 1:1000). The membranes were subsequently washed, then incubated for 60 min at room temperature with suitable HRP-conjugated secondary antibodies (dilution: 1:5000). The ECL reagent kit and gel imaging equipment (Bio-Rad, Hercules, CA, USA) were used to detect the presence of proteins on the membrane.

### 2.9. Histopathology Studies

Kidney tissues were post-fixed in 4% formaldehyde, then processed for paraffin sectioning and staining. A microtome was used to cut paraffin sections at a thickness of 3 µm. The cut sections were then treated to remove the wax, and stained using hematoxylin plus eosin (H&E) dye for histopathological examination. Kidney histology images were acquired using a DP72 camera coupled to an Olympus BX microscope.

### 2.10. Data Analysis

The data were analyzed with the computer-based program Graph Pad Prism 5 (San Diego, CA, USA), and the results are presented as means and standard deviations (mean ± SD). Between-group differences were evaluated using one-way ANOVA followed by Tukey’s comparison test, with a *p*-value of less than 0.05 indicating statistical significance (*p* < 0.05).

## 3. Results

### 3.1. Geraniol Protects Kidneys against Doxorubicin-Mediated Injury

**Figure 1:**
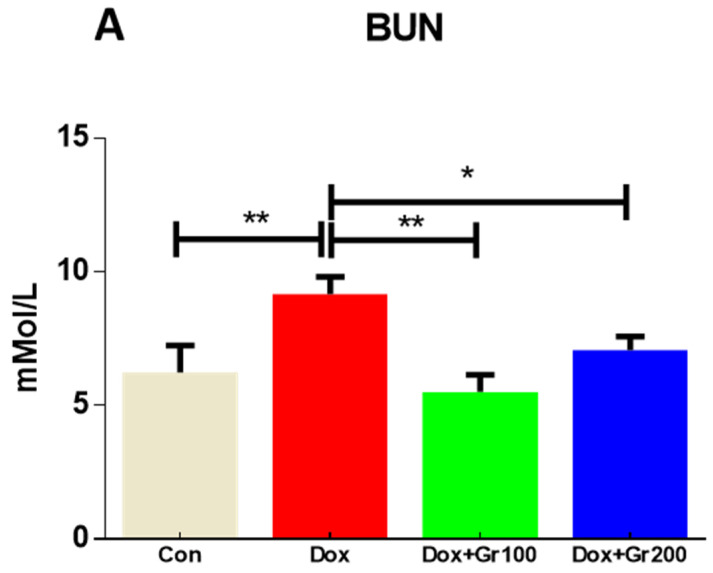

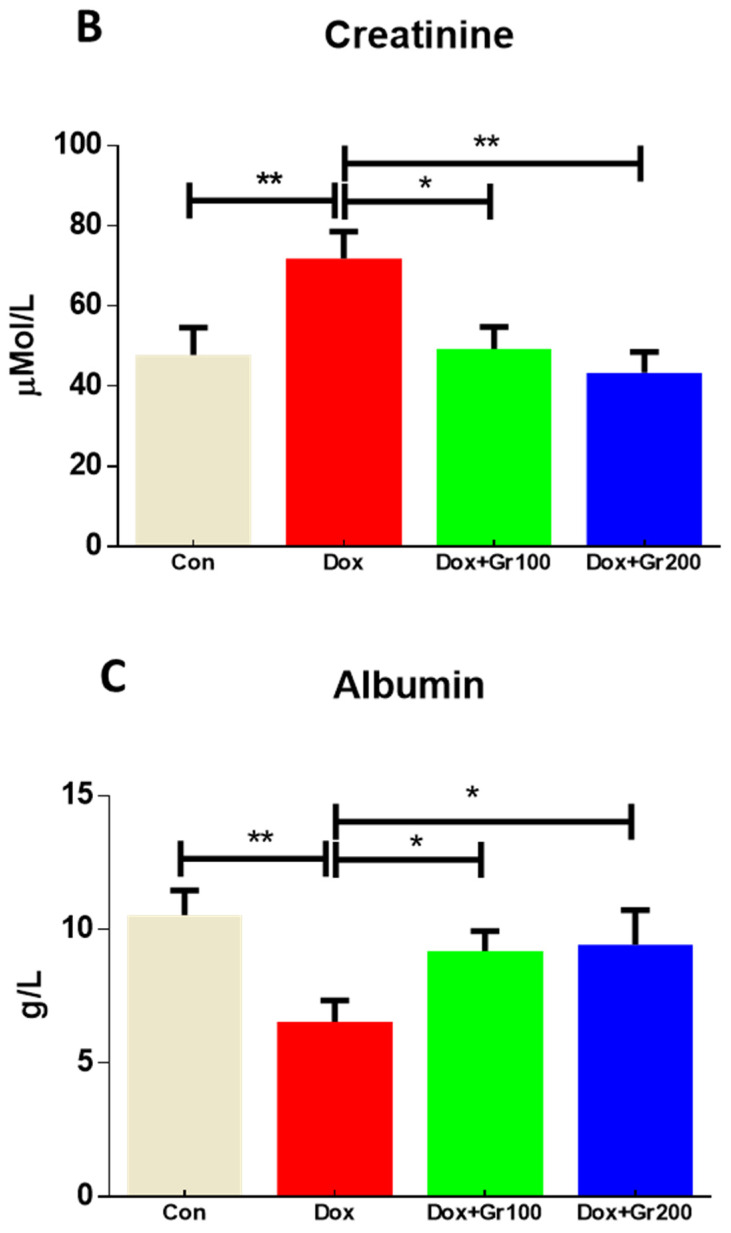
(**A**–**C**) Effects of geraniol (Gr), doxorubicin (Dox), or their combination (Dox + Gr100 and Dox + Gr200) on kidney function markers. Data are presented as mean + SD (n = 5). *, *p* < 0.05; **, *p* < 0.01; Gr100 and Gr200 indicate 100 and 200 mg of geraniol per kilogram of body weight, respectively.

This study examined blood creatinine, albumin, and BUN levels to determine whether Dox administration caused renal injury. As expected, Dox treatment caused significant increases in BUN and creatinine, alongside a significant reduction in albumin (Figure 1A–C). Geraniol, pre-treatment, ameliorated the observed abnormalities in creatinine, albumin, and BUN levels, indicating that it may protect against Dox-mediated renal injury.

### 3.2. Geraniol Protects against Doxorubicin-Mediated Oxidative Stress

**Figure 2:**
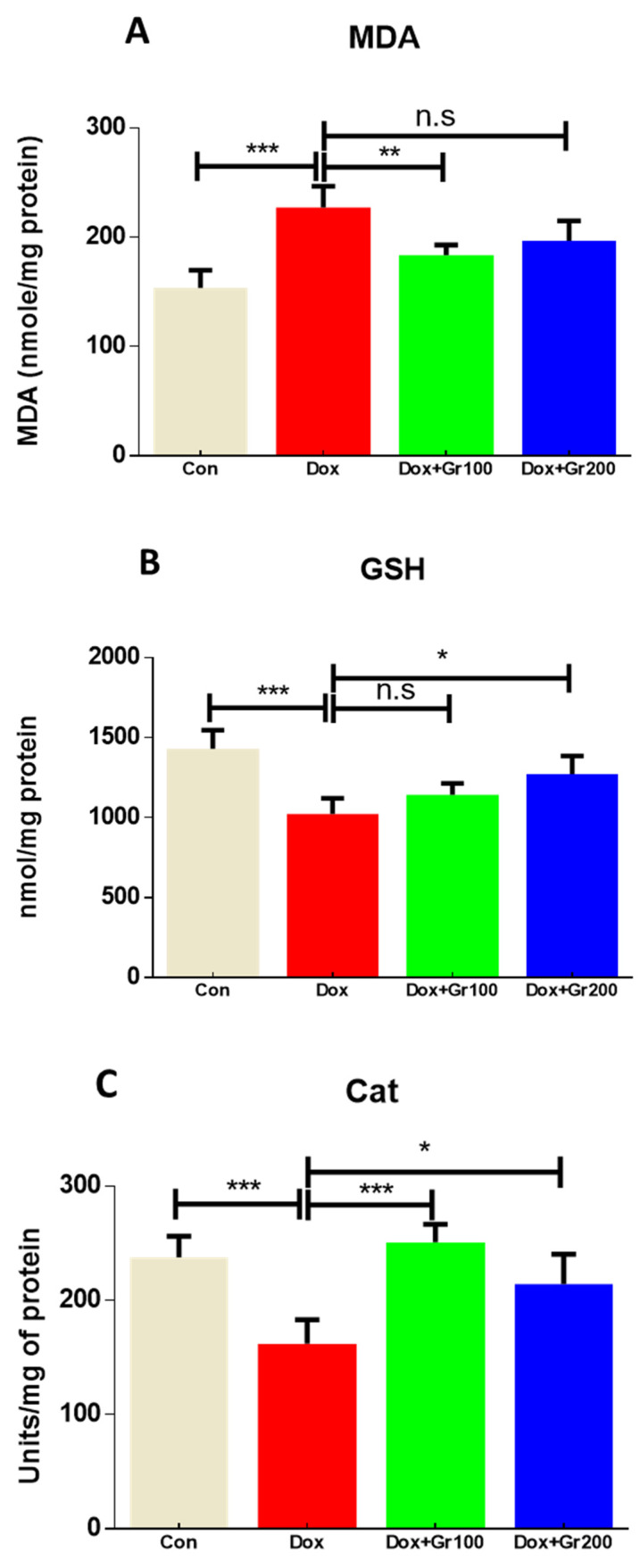
(**A**–**C**) Effects of geraniol (Gr), doxorubicin (Dox), or their combination (Dox + Gr100 and Dox + Gr200) on kidney oxidative stress indicators. Data are presented as mean + SD (n = 5). *, *p* < 0.05; **, *p* < 0.01; ***, *p* < 0.001; n.s., *p* > 0.05 or non-significant. Gr100 and Gr200 indicate 100 and 200 mg of geraniol per kilogram of body weight, respectively.

**Figure 3:**
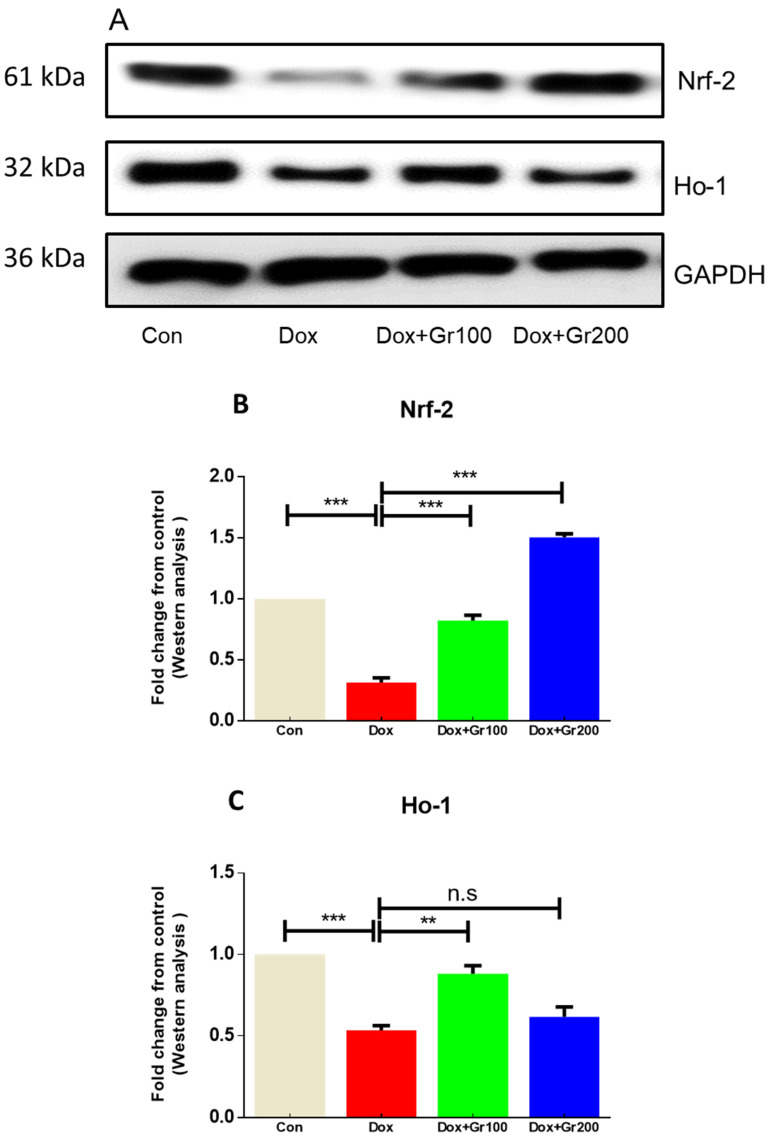

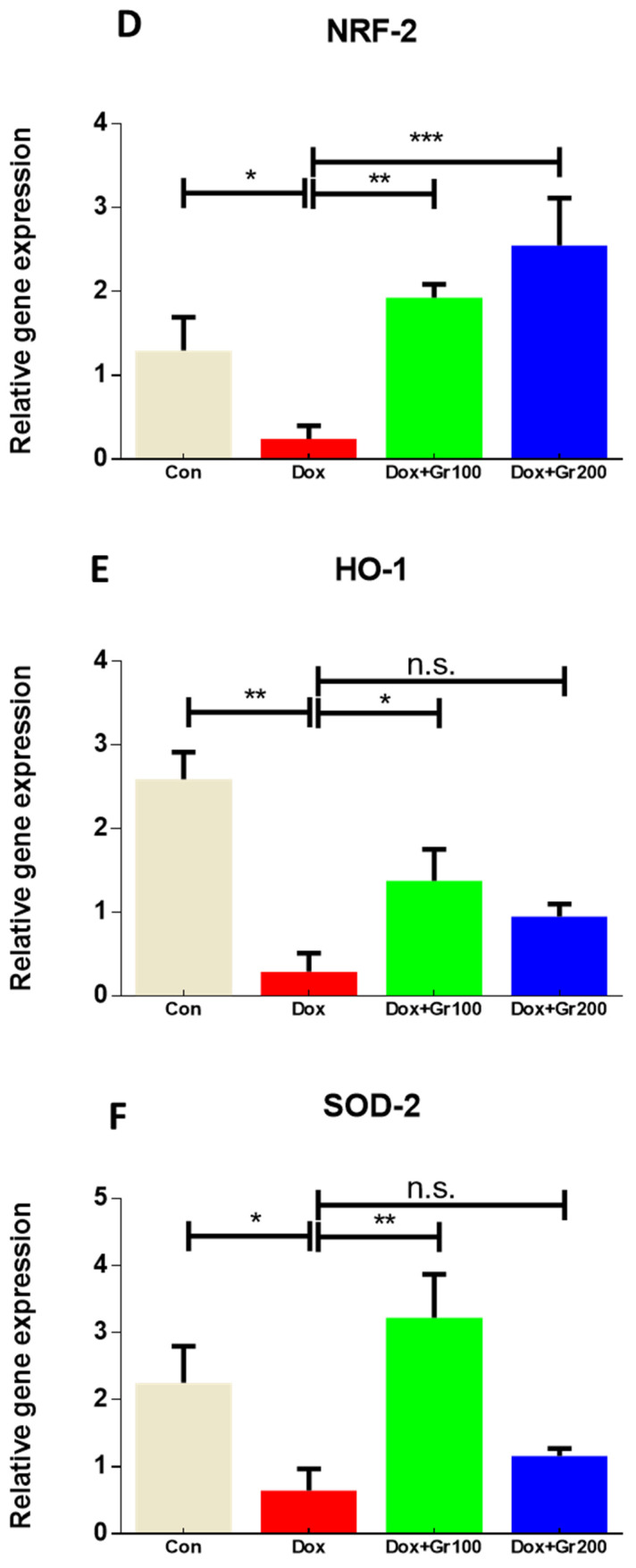

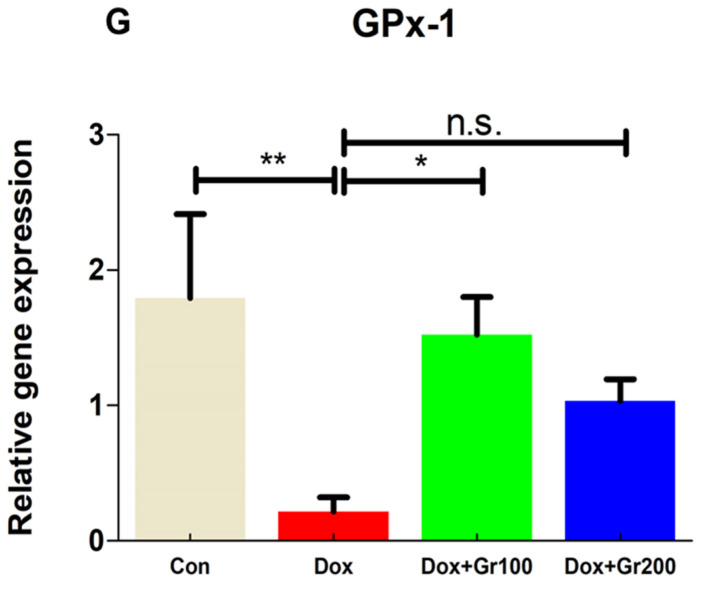
(**A**–**G**) Effects of geraniol (Gr), doxorubicin (Dox), or their combination (Dox + Gr100 and Dox + Gr200) on protein and gene expression of antioxidant stress markers. Data are presented as mean + SD (n = 6). *, *p* < 0.05; **, *p* < 0.01; ***, *p* < 0.001; n.s., *p* > 0.05 or non-significant. Gr100 and Gr200 indicate 100 and 200 mg of geraniol per kilogram of body weight, respectively.

To validate if geraniol supplementation may reduce Dox-associated oxidative stress and improve antioxidant capacity, we quantified the amount of MDA and GSH, the activity of CAT, and the gene and protein expression of Nrf-2, Ho-1, SOD-2, and GPx-1 in kidney tissue. Compared with the control group, a single Dox injection (20 mg/kg) resulted in a substantial rise of MDA along with significant reductions in GSH content and CAT activity (Figure 2A–C). It also significantly decreased the gene and protein expression of Nrf-2, Ho-1, GPx-1, and SOD-2 (Figure 3A–G). However, prophylactic supplementation with geraniol substantially recovered the modifications of these parameters in a dose-dependent manner. These findings reveal the potential antioxidant action of geraniol.

### 3.3. Geraniol Protects against Doxorubicin-Mediated Kidney Inflammation

**Figure 4:**
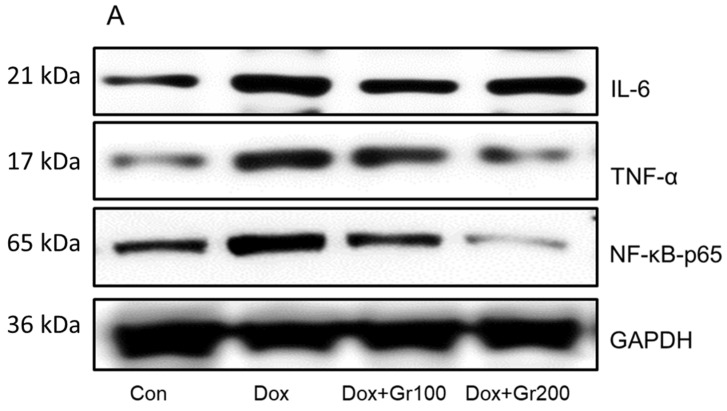

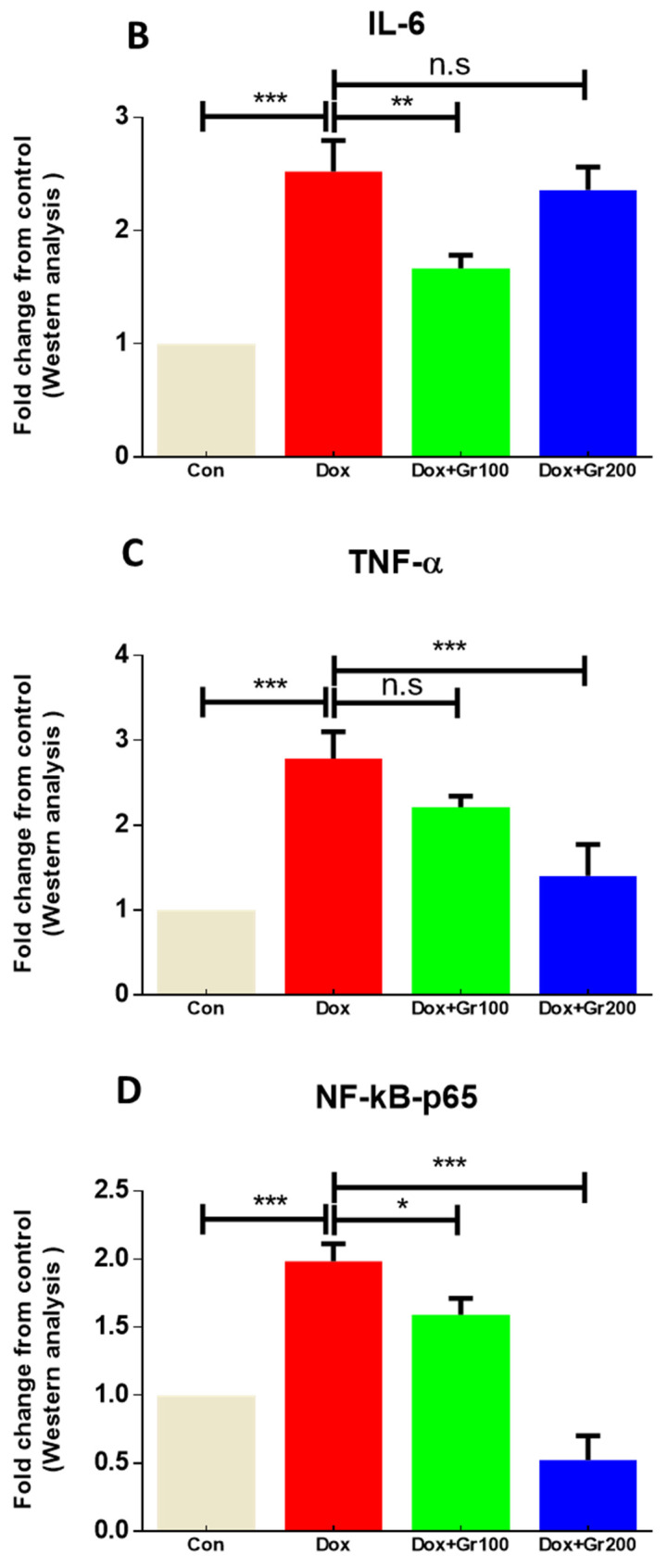
(**A**–**D**) Effects of geraniol (Gr), doxorubicin (Dox), or their combination (Dox + Gr100 and Dox + Gr200) on protein expression of inflammatory mediators. Data are presented as mean + SD (n = 6). *, *p* < 0.05; **, *p* < 0.01; ***, *p* < 0.001; n.s., *p* > 0.05 or non-significant. Gr100 and Gr200 indicate 100 and 200 mg of geraniol per kilogram of body weight, respectively.

Geraniol’s anti-inflammatory activities have been demonstrated elsewhere. To further confirm the potential anti-inflammatory action of geraniol in the context of Dox treatment, we used Western blots to evaluate the expression of proteins important in inflammatory regulation (NfkB-p65, TNF-α, and IL-6). The results showed that expression changes driven by Dox were restored in the rats treated with geraniol (Figure 4A–D), and indicate that geraniol has anti-inflammatory effects.

### 3.4. Geraniol Protects against Doxorubicin-Mediated Apoptosis

**Figure 5:**
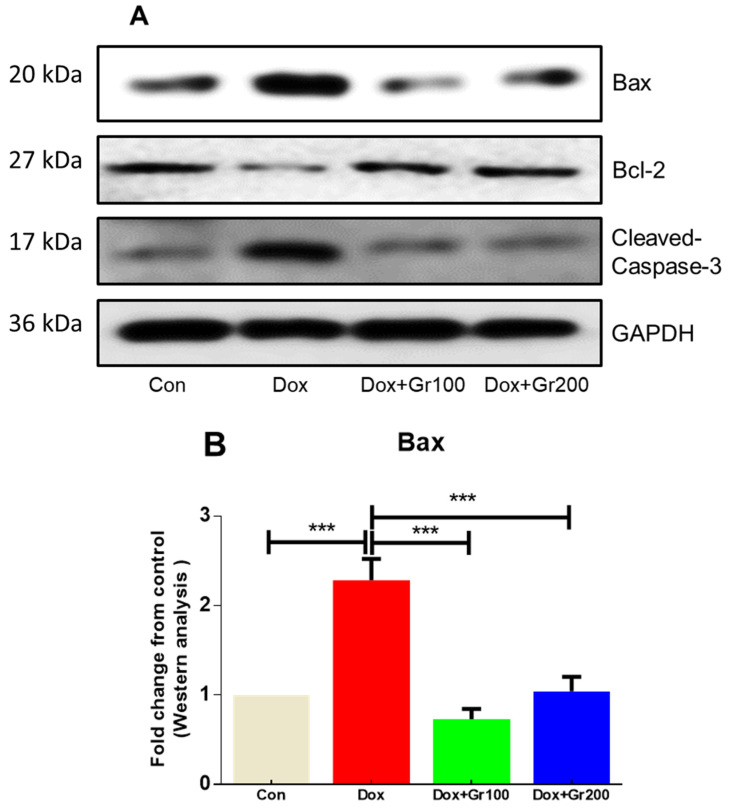

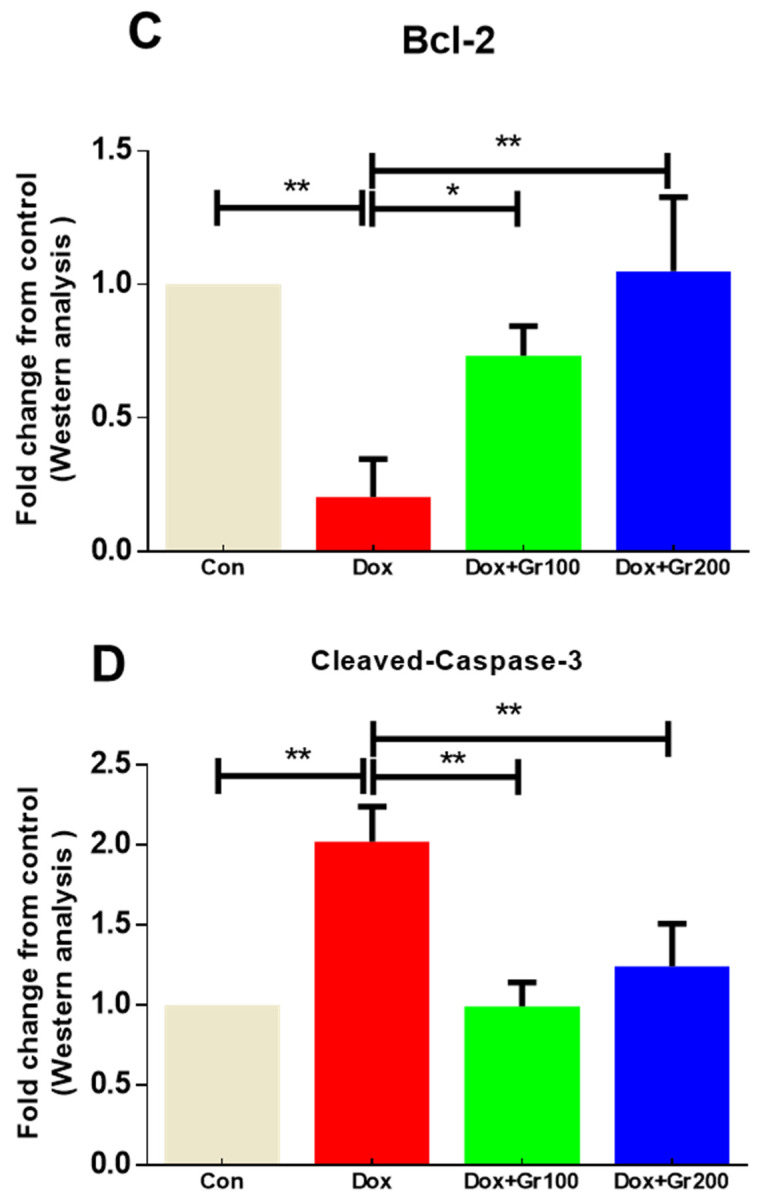
(**A**–**D**) Effects of geraniol (Gr), doxorubicin (Dox), or their combination (Dox + Gr100 and Dox + Gr200) on protein expression of apoptotic markers by Western blot. Data are presented as mean + SD (n = 6). *, *p* < 0.05; **, *p* < 0.01; ***, *p* < 0.001. Gr100 and Gr200 indicate 100 and 200 mg of geraniol per kilogram of body weight, respectively.

To examine whether geraniol suppresses apoptosis induced by Dox, we measured the expression of pro-apoptotic and anti-apoptotic protein markers. When compared with the control group, a single dosage of Dox led to a significant increase in the renal tissue expression of pro-apoptotic proteins (Bax and cleaved caspase-3) and substantial decline in the levels of the anti-apoptotic protein Bcl-2 (Figure 5A–D). Meanwhile, supplementation with geraniol rectified those abnormalities. These findings suggest that pre-treatment with geraniol reduces Dox-mediated apoptosis in the rat kidney.

### 3.5. Geraniol Protects against Doxorubicin-Mediated Alteration in Kidney Architecture

**Figure 6:**
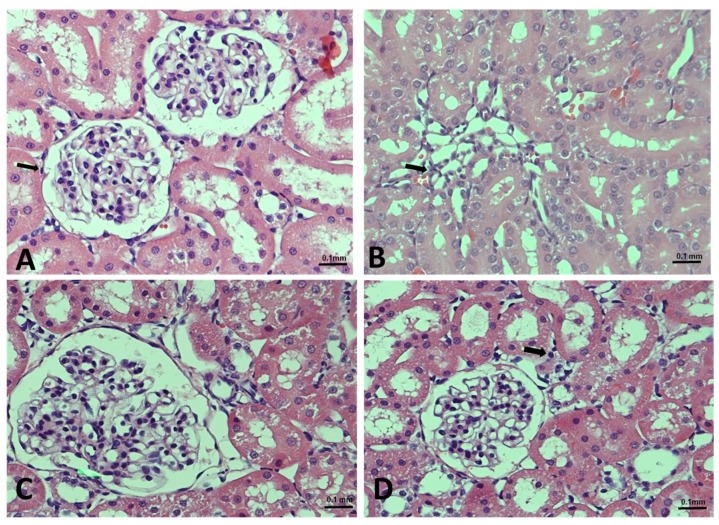
(**A**–**D**). Effects of geraniol (Gr), doxorubicin (Dox), or their combination (Dox + Gr100 and Dox + Gr200) on kidney tissue as visualized using H&E staining and light microscopy: (**A**) normal architecture of the kidney; (**B**) mild interstitial inflammation (indicated by arrows) consisting of lymphocytes, caused by Dox administration; and (**C**,**D**) geraniol-treatment-improved glomeruli, tubules, and tubular epithelial cell morphology at both tested doses. Scale bar is 0.1 mm.

Finally, to further confirm our results, we examined the histopathology of renal tissue. Rats in the control group had normal tubules and glomeruli (Figure 6A), while those with administered Dox exhibited glomerular congestion and tubular destruction (Figure 6B). We found that supplementation with geraniol restore the Dox-mediated damage in a dose-dependent manner (Figure 6C,D).

## 4. Discussion

Chemotherapeutic medicines are frequently employed to treat different types of cancer; however, these drugs often also destroy physiological homeostasis in numerous organs and can lead to physiological adverse effects in non-tumor cells, mostly due to free radical formation and oxidative stress toxicity [42]. The goal of this investigation was to determine whether geraniol could protect Wistar rats against Dox-mediated kidney damage. Accordingly, we investigated Dox-mediated inflammation, apoptosis, and oxidative stress, and evaluated whether geraniol can act as a preventive agent.

Albumin, creatinine, and blood urea nitrogen (BUN) are reliable indicators for kidney damage [11]. Normal renal tissue does not permit albumin to flow out from the bloodstream to the urine, while also filtering creatinine and BUN from the bloodstream into the urine. However, in the event of renal dysfunction, these processes are disturbed: albumin is excreted in the urine, resulting in reduced serum concentrations, and creatinine and BUN are not filtered correctly, resulting in elevated serum values [11]. In the current work, Dox treatment caused increases in serum creatinine and BUN alongside a significant decrease in albumin. The results are in fair agreement with the published reports [11]. We also found prophylactic supplementation with geraniol to attenuate these changes, suggesting that geraniol may prevent renal damage caused by Dox.

Lipid peroxidation is a hallmark of oxidative stress, and many investigations have shown that Dox administration leads to increased levels of malonaldehyde (MDA), a product of lipid peroxidation [7,43]. We confirmed that Dox caused a significant rise in MDA levels within kidney tissue and, moreover, that pre-supplementation with geraniol significantly and dose-dependently reduced this effect. Younis and colleagues, likewise, demonstrated that geraniol substantially reduces MDA levels in methotrexate-mediated kidney injury [44]. These results suggest that geraniol may be effective in treating Dox-mediated kidney damage due to its potent inhibition of lipid peroxidation.

Living organisms utilize various enzymatic and non-enzymatic antioxidants to remove free radicals, thereby offering an effective defense against ROS. Essential anti-oxidative agents include the non-enzymatic antioxidant GSH and its oxidized analogs; GSH interacts directly with free radicals via its -SH group [45]. In the current study, a single Dox injection demonstrably reduced the GSH reservoir, and geraniol pre-treatment was able to reverse this effect. These findings are supported by previously published studies [46,47].

ROS production within cells is also linked to depletion of anti-oxidative enzymes. In particular, the enzymes CAT and glutathione reductase (GR) accelerate conversion of H_2_O_2_ and other ROS to H_2_O and O_2_, while SOD assists in converting superoxide anion free radicals to H_2_O_2_, which is subsequently removed by CAT or GPx [45]. We found that expression of all examined antioxidant enzymes, namely SOD, CAT, and GPx-1, was statistically reduced in the Dox group compared with the control group. However, geraniol pre-administration rescued the Dox-induced reduction of SOD, GPx-1, and CAT levels, probably by scavenging ROS through its own antioxidant ability.

Various enzymes that protect cells from harmful oxidative stress have been linked to the Nrf2 pathway, and stimulating the Nrf2/Ho-1 pathway has been shown to considerably improve kidney function [21,48,49]. To mechanistically validate the protective effect of geraniol, we examined Nrf2 and Ho-1 levels in relation to Dox administration. We found levels of these proteins to be significantly lower in the Dox group than in the controls, consistent with prior studies [20,21]. Meanwhile, geraniol supplementation prevented Dox-associated kidney damage by boosting mRNA and protein expression of members of the Nrf2/Ho-1 signaling pathway, which is in line with the findings of Younis et al. [44].

Ultimately, inflammation is one of the most likely causes of Dox-mediated kidney injury. Induction of the nuclear factor kappa B (NF-kB) pathway is known to play a very important role in the pathophysiology of Dox-mediated kidney inflammation [50], with NF-kB being a transcription factor that regulates expression of several genes associated with inflammation [51], such as those encoding TNF-α, IL-1β, and IL-6 [52,53]. Here, Dox-treated animals were found to exhibit increased levels of TNF-α, IL-6, and NfkB-p65 [54], while geraniol pre-supplementation reduced these inflammatory mediators in a dose-dependent manner. These data suggest that geraniol’s anti-inflammatory action may be due to an inhibitory effect against the NF-kB pathway.

Another critical player in the etiology of Dox-mediated nephrotoxicity is apoptosis, which is regulated by the balance of pro-apoptotic and anti-apoptotic proteins. Caspase-3 and Bax are pro-apoptotic proteins that increase the porosity of the mitochondrial membrane and enable cytochrome c to leak from the intermembrane gap, triggering apoptosis through the intrinsic apoptotic pathway. In contrast, Bcl-2 is an anti-apoptotic protein located in the outer mitochondrial membrane that helps maintain the mitochondrial structure and inhibits cytochrome c leakage into the cytoplasm, preventing apoptosis. The ratio of Bax and Bcl-2 thus affects cell survival [11,24,25,26,55]. In the current work, Dox treatment resulted in significant elevations of cleaved-caspase-3 and Bax alongside a dramatic reduction in expression of Bcl-2, a pattern reported in the published literature [11]. Geraniol pre-supplementation significantly mitigated this Dox-associated alteration in the balance of apoptotic proteins.

Finally, we performed a histopathological examination to investigate whether Dox administration causes renal damage. This examination confirmed that Dox administration leads to the congestion of the renal blood vessels, interstitial inflammation involving lymphocytes, and hemorrhage between the tubules. Meanwhile, geraniol pre-supplementation clearly alleviated the tissue damage caused by Dox.

**Figure 7:**
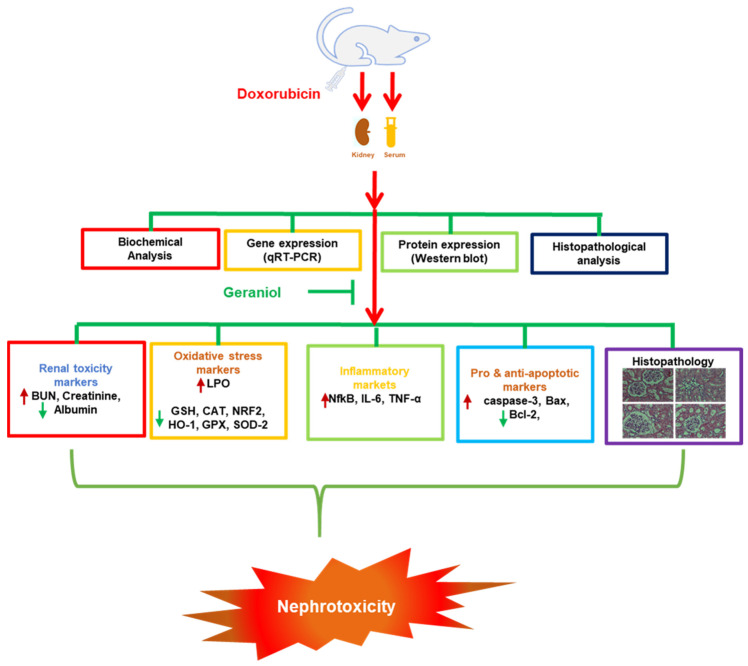
Diagram of the proposed nephroprotective mechanism of geraniol against the toxic effects of doxorubicin.

In conclusion, the data obtained from the present investigation demonstrate, for the first time, the beneficial effects of geraniol against Dox-mediated kidney toxicity, likely realized by lowering oxidative stress, inflammation, and apoptotic tissue damage through the modulation of the NF-κB, Bax/Bcl-2, and Nrf2/Ho-1pathways (Figure 7). However, the exact protective mechanism of geraniol is yet to be elucidated; further study remains required to pinpoint the details of its action.

## Figures and Tables

**Table 1:** The primer sequence utilized in this study.

Gene	Primer Sequences (5′→3′) Forward	Primer Sequences (5′→3′) Reverse	Reference
NRF2	CACATCCAGACAGACACCAGT	CTACAAATGGGAATGTCTCTGC	Manually designed
HO-1	ACAGGGTGACAGAAGAGGCTAA	CTGTGAGGGACTCTGGTCTTTG	Manually designed
SOD-2	TTCGTTTCCTGCGGCGGCTT	TTCAGCACGCACACGGCCTT	Manually designed
GPx-1	AGTTCGGACATCAGGAGAATGGCA	TCACCATTCACCTCGCACTTCTCA	Manually designed
GAPDH	TCTGCTCCTCCCTGTTCTAGAGACA	TTGTGAGGGAGATGCTCAGTGTTGG	Manually designed

## Data Availability

All the information gathered has been incorporated into the paper.
